# The Influence of Accelerated UV-A and Q-SUN Irradiation on the Antimicrobial Properties of Coatings Containing ZnO Nanoparticles

**DOI:** 10.3390/molecules22091556

**Published:** 2017-09-17

**Authors:** Małgorzata Mizielińska, Łukasz Łopusiewicz, Monika Mężyńska, Artur Bartkowiak

**Affiliations:** Center of Bioimmobilisation and Innovative Packaging Materials, Faculty of Food Sciences and Fisheries, West Pomeranian University of Technology Szczecin, Janickiego 35, Szczecin 71-270, Poland; lukasz.lopusiewicz@zut.edu.pl (Ł.Ł.); monika.mezynska@zut.edu.pl (M.M.); artur.bartkowiak@zut.edu.pl (A.B.)

**Keywords:** coatings, ZnO nanoparticles, antibacterial, antimicrobial properties

## Abstract

The influence of accelerated UV-A and Q-SUN irradiation on the antimicrobial properties of coatings containing ZnO nanoparticles was investigated using a polyethylene (PE) film covering. The results of the study showed that Methyl Hydroxypropyl Celluloses (MHPC) coatings did not influence the growth of *S*. *aureus*, *B*. *cereus*, *E*. *coli*, *P*. *aeruginosa* or *C*. *albicans* cells. MHPC coatings containing ZnO nanoparticles inhibited the growth of bacterial strains and reduced the number of *C*. *albicans* strains. Accelerated Q-SUN and UV-A irradiation had no influence on the antimicrobial effect of nano ZnO coatings against *S*. *aureus*, *B*. *cereus* and *E*. *coli*. Q-SUN irradiation decreased the activity of MHPC coatings containing nanoparticles against *P*. *aeruginosa* and *C*. *albicans*. An FT-IR analysis clearly showed that ZnO nanoparticles shielded the MHPC coating during Q-SUN irradiation.

## 1. Introduction

The use of active antimicrobial compounds incorporated in packaging material has begun to receive more attention, for its use as control agents against bacteria in food packaging systems. It can ensure microbial food safety for the consumer, and be invaluable for the extension of product shelf life [1,2,3]. The contact between active materials and food, which has the ability to change food’s composition or the atmosphere around it, represents an active packaging system that inhibits the growth of microorganisms present on the surface of food products [1,2,4]. Zinc Oxide (ZnO) nanoparticles have been explored as antimicrobial agents, used in active food packaging systems, as one of five various zinc compounds that are regarded as being safe (GRAS) by the United States food and drug administration (USFDA, 21CFR182.8991) [1,2,5]. Zinc Oxide nanoparticles offer bactericidal effects for Gram-positive and Gram-negative bacteria, to spores that are resistant to high temperature, high pressure [6,7,8], yeasts and moulds [9]. Zinc Oxide nanoparticles have been added to petroleum-derived polymers such as LDPE, PP, PU or PET using conventional incorporation methods, such as melt mixing or solvent casting, as well as being added to biodegradable polymers such as PHA [10]. The nanoparticles were also introduced into polymer coating layers, in the application of antimicrobial packaging [2,11]. Numerous studies have shown an increase in the shelf life of food products packed in films containing ZnO nanoparticles (within a polymer matrix), or used with coatings containing ZnO nanoparticles [9,12,13,14,15]. The shelf life of sliced wheat bread was extended from 3 to 35 days using packaging containing nanoparticles, as compared to control versions. All active coatings reduced the number of yeasts and moulds in sliced bread for 15 days, and offered a further improvement in antimicrobial properties obtained for active coatings, with no fungal growth over a 15-day period [9]. Films containing nano-ZnO exhibited excellent antimicrobial activity and were fabricated into packaging pouches for raw meat. The prepared pouches showed significant action against microbes in the raw meat, owing to their complete inhibition of microbial growth, to the sixth day of storage at 4 °C [12].

Polyethylene (PE) is widely used in food packaging due to its flexibility, transparency, thermo stability and low cost [16]. To create antimicrobial properties, PE films can be covered with active coatings containing ZnO nanoparticles [2,3]. In general, an active packaging material should function during storage to inhibit bacterial, yeast, and mould growth to extend the shelf life of the food product. This means that coatings should offer sufficient resistance against ultraviolet (UV) radiation or be shielded against UV. Ultraviolet radiation is a part of the non-ionizing region of the electromagnetic spectrum, which comprises approximately 8–9% of total solar radiation. It can lead to a degradation in the physico-mechanical, optical and antimicrobial properties of materials. Introducing an active substance that is sensitive to UV in a coating carrier can lead to an inactivation of the coating after UV-aging. Introducing an active substance that is resistant to UV in a coating carrier, or adding a substance with shielding properties, can prevent an inactivation of the coating after UV-aging. The rapid development of nanotechnology has resulted in the implementation of ZnO nanoparticles in coatings, to enhance the properties of such coatings without a significant influence on their transparency. Additionally, nanoparticles have attracted great interest, and the development of coating applications as agents to improve anticorrosion properties has increased, particularly as UV absorbers. It was shown that ZnO nanoparticles exhibited superior chemical stability under UV radiation, compared to other organic UV absorbers. The application of nanoparticles can improve UV-shielding of any respective packaging film materials. Nano-ZnO particles can even protect their own antimicrobial properties [17,18,19,20].

The purpose of this research was to study the influence of accelerated UV-A and Q-SUN irradiation (UV-aging) on the antimicrobial properties of coatings containing ZnO nanoparticles.

## 2. Results

### 2.1. Antimicrobial Properties

**Figure 1:**
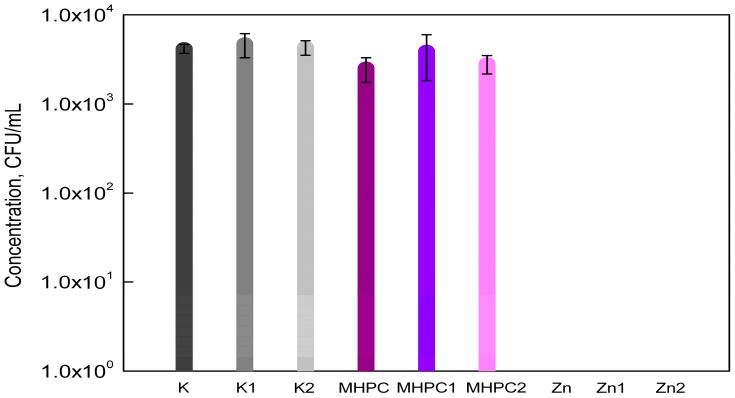
The influence of coatings on *S*. *aureus* growth. K—PE film; K1—UV-A irradiated PE film; K2—Q-SUN irradiated PE film; MHPC—PE film, covered with MHPC coating; MHPC1—UV-A irradiated PE film, covered with MHPC coating; MHPC2 Q-SUN irradiated PE film, covered with MHPC coating; Zn—PE film, covered with MHPC coating, containing ZnO nanoparticles; Zn1—UV-A irradiated PE film, covered with MHPC coating, containing ZnO nanoparticles; Zn2 Q-SUN irradiated PE film, covered with MHPC coating, containing ZnO nanoparticles.

The results of the study showed that MHPC coatings did not have an influence on the growth of *S*. *aureus* cells, as was indicated by a previous study [2]. The MHPC coatings containing ZnO nanoparticles inhibited the growth of *S*. *aureus*. The accelerated Q-SUN and UV-A irradiation did not influence the antimicrobial properties of the coatings with nano-ZnO. In the case of MHPC coatings devoid of nanoparticles, the number of bacterial cells increased for films that were irradiated with UV-A (Figure 1). An increase in the number of the *S*. *aureus* was observed from 2.53 × 10^3^ to 3.90 × 10^3^ (CFU/mL). Statistical analysis showed that the increase in the number of bacterial cells was not significant (*p* > 0.05).

**Figure 2:**
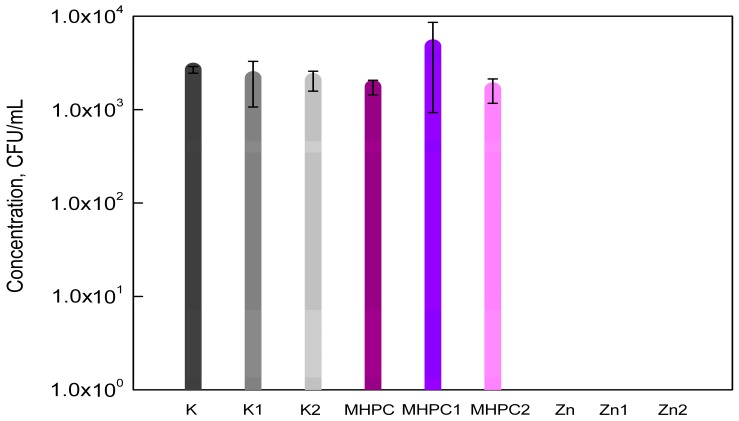
The influence of coatings on *B*. *cereus* growth.

The susceptibility assay of *B*. *cereus* with respect to the active coatings containing nano-ZnO is shown in Figure 2. The results of this research determined that MHPC coatings were not found to be active against bacteria. The *B*. *cereus* cells exhibited sensitivity towards coatings containing ZnO nanoparticles. Q-SUN and UV-A irradiation did not deactivate the antimicrobial properties of coatings with nano-ZnO. An increase in the number of bacterial cells for MHPC coatings irradiated with UV-A was observed. The differences between the numbers of viable cells were significant, as confirmed by a Duncan test (*p* < 0.01).

**Figure 3:**
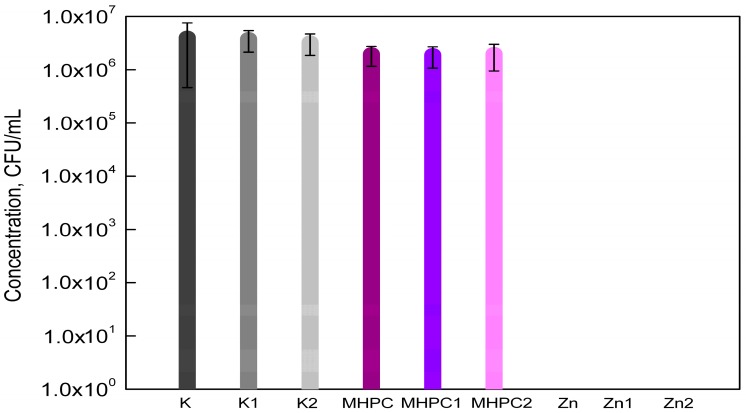
The influence of coatings on *E*. *coli* growth.

The results of this research demonstrated that MHPC coatings had no influence on the growth of *E. coli* cells. These results were confirmed by a previous study [2]. The growth of bacterial cells after 24 h contact with MHPC coatings containing ZnO nanoparticles was not observed. As emphasised below [Figure 3], the influence of accelerated Q-SUN and UV-A irradiation on the antimicrobial properties of coatings with nano ZnO was also not seen. The statistical analysis demonstrated that the differences between numbers of *E. coli* cells were not significant (*p* > 0.05).

**Figure 4:**
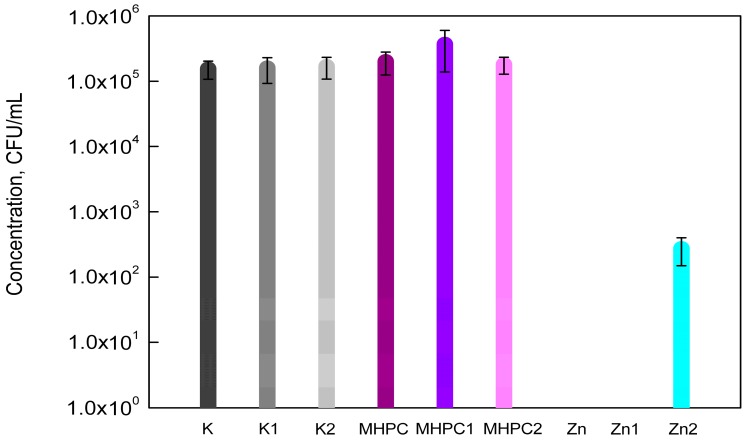
The influence of coatings on *P*. *aeruginosa* growth.

It was demonstrated in this study that MHPC coatings did not decrease the growth of *P*. *aeruginosa* strain (*p* > 0.05). Zinc Oxide nanoparticles as an additive to the coating completely inhibited the growth of bacterial cells. As can be seen in Figure 4, UV-A irradiation did not influence the antimicrobial properties of coatings with nano-ZnO. As previously shown, Q-SUN irradiation decreased the activity of the MHPC coatings containing nanoparticles. The growth of *P*. *aeruginosa* was observed, but the number of bacterial cells was more reduced than in the case of PE films devoid of coating, or MHPC coating devoid of nano-ZnO. It was observed that the number of *P*. *aeruginosa* cells decreased from 1.70 × 10^5^ (K2) and 1.81 × 10^5^ (MHPC2) to 2.75 × 10^2^ (CFU/mL) (Zn2). As indicated by statistical analysis, the decrease in the number of bacterial cells was significant (*p* < 0.01—differences between K2 and Zn2; and between MHPC2 and Zn2).

**Figure 5:**
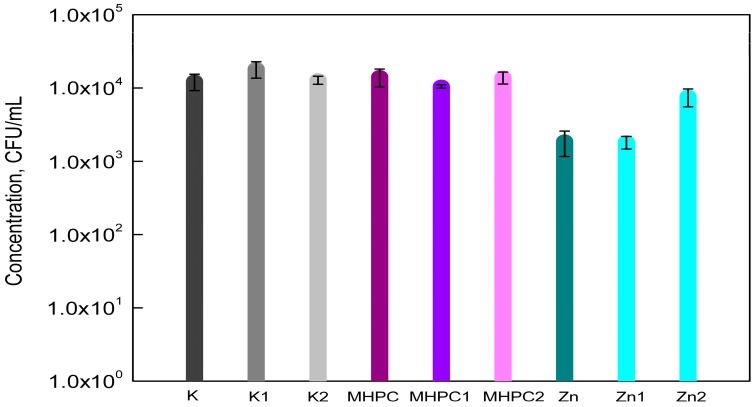
The influence of coatings on *C*. *albicans* growth.

The results of this study showed that MHPC coatings did not influence the growth of *C*. *albicans* cells, compared to PE films that were not covered. The MHPC coatings containing ZnO nanoparticles reduced the growth of viable cells. It was determined that the number of *C*. *albicans* cells decreased from 1.23 × 10^4^ (K) and from 1.42 × 10^4^ (MHPC) to 1.88 × 10^3^ (CFU/mL). A Duncan’s test confirmed that the influence of nano-ZnO on antimicrobial properties of (K) PE films (*p* < 0.001) or PE films covered with MHPC (*p* < 0.001) was significant. It was shown [21] that zinc is effective in inhibiting the growth of *C*. *albicans*. It would appear that nanoparticle size and shape play a vital role in antimicrobial activity. The cellular membranes in the microorganism cells contain pores with a diameter measured in nanometers. Considering that nanoparticles are smaller than the microorganism pores, they have the unique property of crossing the cell membrane without hindrance [22,23]. Accelerated UV-A irradiation had no influence on the antimicrobial properties of coatings with nano-ZnO (Figure 5). This was confirmed by a Duncan test (*p* > 0.05). A log reduction of the number of *C*. *albicans* cells was also noted. A decrease in the number of viable cells was observed for coating with nanoparticles irradiated with Q-UV. It was shown that Q-UV irradiation had a clear influence, decreasing the antimicrobial properties of the coating. The decrease in antimicrobial activity was significant (*p* < 0.001).

### 2.2. FT-IR Analysis

**Figure 6:**
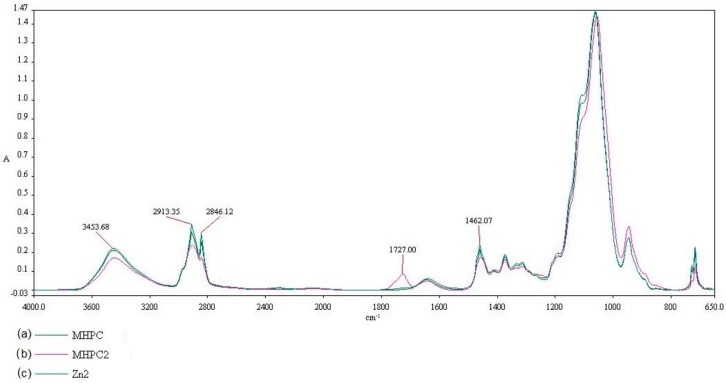
The FT-IR spectra of (a) MHPC coating (b) Q-SUN irradiated MHPC coating and (c) Q-SUN irradiated MHPC coating with ZnO nanoparticles

The influence of UV irradiation and Q-SUN irradiation on coatings can be clearly noted using Fourier transform infrared (FT-IR) spectroscopy. The properties that influence the absorption peak and band positions are the structure, the chemical composition, and the morphology of thin films [17]. The results of this study demonstrated that differences in chemical composition and morphology of PE films (K) after UV-A irradiation (K1) and Q-SUN irradiation (K2) were not found. The results indicated that accelerated irradiation had no effect on PE film samples. The influence of UV-A irradiation on MHPC (MHPC1) or MHPC coatings containing ZnO nanoparticles (Zn1) was also not observed. A MHPC coating (MHPC), a Q-SUN irradiated MHPC coating (MHPC2) and a Q-SUN irradiated MHPC coating with ZnO nanoparticles (Zn2) are presented in Figure 6. There are four regions viewed in the FT-IR spectroscopy, extending between (1) ranges from 3600 to 3200 cm^−1^; (2) ranges from 3200 to 2800 cm^−1^; (3) ranges from 1800 to 1600 cm^−1^ and (4) ranges from 1600 to 1400 cm^−1^. In the case of a 3453.68 cm^−1^ peak, a peak consistency with absorption can be noted, stimulated by O-H single bonds. Alternatively, spectra peaks at 2913.35, 2846.12 and 1462.07 cm^−1^, can be observed for a peak with CH_3_-CH_2_ induced absorption. Different peak properties were shown, ranging from 1800 cm^−1^ to 1600 cm^−1^. A 1727.00 cm^−1^ peak was not observed in the case of the MHPC coating. The presence of this peak for a Q-SUN irradiated MHPC coating was noted, simulated by C=O double bonds. It was clearly proven that accelerated Q-SUN irradiation altered the chemical composition of the MHPC layer. A 1727.00 cm^−1^ peak was not observed for Q-SUN irradiated MHPC coatings containing ZnO nanoparticles. It is tempting to suggest that nano-ZnO shielded the MHPC layer against Q-SUN irradiation. This conclusion was indicated by El-Feky O.M. et al. [19] who used ZnO nanoparticles as an additive for a coating created for oil paints on paper, to protect them against UV irradiation.

### 2.3. Scanning Electron Microscopy (SEM)

**Figure 7:**
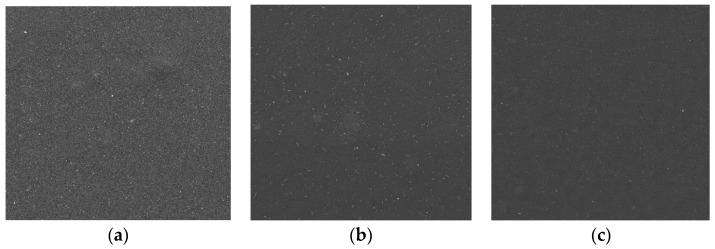
The SEM images of (**a**) MHPC coating (**b**) Q-SUN irradiated MHPC coating and (**c**) Q-SUN irradiated MHPC coating with ZnO nanoparticles.

Scanning electron microscopy (SEM) is the most widely applied technique to characterize the shape, size, morphology, and porosity of matrices [17]. Figure 7 shows SEM images of coatings containing ZnO nanoparticles. SEM images revealed that ZnO nanoparticles were homogeneously distributed throughout the coating surfaces. Differences between the control coating and the irradiated coatings were not observed.

## 3. Discussion

Nano-Zinc Oxide (ZnO) particles exhibit many advantages, such as low cost, UV blocking properties and a white appearance [2,20]. Moreover, many studies have indicated a highly specific toxicity of zinc oxide nanoparticles against bacteria, yeast, moulds, and a non-toxicity to human cells [24]. El-Feky O.M. et al. [19] introduced ZnO nanoparticles into a coating for oil paints on paper, which protected them against UV aging and microbial attack. Regarding antifungal activity, ZnO nanoparticles showed significant antifungal activity against some fungal strains, such as *Aspergillus flavus*, *A*. *niger* and *Candida albicans*. Previous studies [2] have demonstrated that ZnO nanoparticles can be introduced into MHPC coatings to cover packaging materials. It has been shown that active coatings exhibited antimicrobial activity against *S*. *aureus* and *E*. *coli* cells, when the growth of the strains was not observed. The goal of our research was to investigate the antimicrobial properties of MHPC coatings with nano-ZnO against *B*. *cereus*, *Pseudomonas aeruginosa* and *Candida albicans*. The influence of UV-aging on antimicrobial properties and the capability of ZnO nanoparticles to protect their own viability were also examined. The results were indicated by a previous study [2] and by Venkatesan R. et al. [25], who indicated that films containing ZnO nanoparticles were highly active in killing *E*. *coli* as Gram-negative and *S*. *aureus* as Gram-positive cells. The antibacterial activity of coatings with nano-ZnO was indicated against both *E*. *coli* and *B*. *atrophaeus* [26]. Aysa N.H. [27] showed antimicrobial properties of ZnO nanoparticles against *P*. *aeruginosa*. The results of the study did not show any difference between the sensitivity of Gram-positive and Gram-negative bacteria towards non-irradiated coatings containing ZnO nanoparticles. The results shown here are not comparable with those reported by Sharma D. et al. [28], who exhibited greater antibacterial activity against *E*. *coli* bacterium than against *S*. *aureus*. Sinha R. et al. [29] and Zhang H. et al. [11] indicated that the nanotoxicity of ZnO nanoparticles was more pronounced on Gram-negative bacteria than on Gram-positive. The opposite results were obtained by Esmailzadeh H. et al. [16] and Gandhi R.R. et al. [30], who indicated that Gram-positive bacteria were more sensitive to ZnO nanoparticles than Gram-negative bacteria were. It is tempting to suggest that the greater sensitivity of Gram-positive compared to Gram-negative bacteria to ZnO nanoparticles is due to differences in cell membrane structure. The cell membrane of the Gram-negative bacteria is composed of lipids, proteins and lipopolysaccharides, whereas the Gram-positive bacteria do not contain lipopolysaccharides and cannot provide effective protection [24,31,32]. Moreover, the small sized ZnO nanoparticles could also permeate the bacterial cell and combine with intracellular DNA and RNA molecules to block genome replication [33]. The suggested mechanism for the antibacterial activity of ZnO nanoparticles is based mainly on the formation of reactive oxygen species (ROS) from water and oxygen [34], which disrupt the integrity of the bacterial membrane—although additional mechanisms have also been suggested.

## 4. Materials and Methods

### 4.1. Materials

The test microorganisms used in this study were obtained from a collection from the Leibniz Institute DSMZ (Deutsche Sammlung von Mikroorganismen und Zellkulturen). The strains were supplied from an American Type Culture Collection (ATCC). The organisms used in this study were *S*. *aureus* strain DSMZ 346, *B*. *cereus* ATCC 14579, *E*. *coli* DSMZ 498, *P*. *aeruginosa* ATCC 27853 and *C*. *albicans* DSMZ 2566.

Polyethylene films, (A4, 50 μm) (KB FOLIE) were used in this research. MHPC (Chempur, Piekary Śląskie, Poland) was used as coating carrier. Zinc Oxide AA 44899, (~70 nm) was used as an active substance. To verify the antimicrobial properties of any coatings, TSB, TSA and Sabouraud mediums (Merck, Darmstadt, Germany) were used. All mediums were prepared according to the Merck protocol (all mediums were weighed according to the manufacturer’s instructions, suspended in 1000 mL of distilled water, and autoclaved at 121 °C for 15 min).

### 4.2. Coating Preparation and Antimicrobial Properties Analysis

(1) 4 g of MHPC was introduced into 100 mL of water. The mixture was mixed for 1 h using a magnetic stirrer (Ika) at 1500 rpm. The mixture was used to cover the PE films to obtain coatings devoid of any active substances.

(2) 0.082 g of ZnO nanoparticles was introduced into 50 mL of water. As a first step, the mixture was mixed for 1 h using a magnetic stirrer (450 rpm). Next, the mixture was sonicated (sonication parameters: cycle: 0.5; amplitude: 20%; time: 10 min), while at the same time, the second mixture (4 g of MHPC into 50 mL) was prepared as described above. The ZnO nanoparticles solution was introduced into the MHPC mixture and sonicated (sonication parameters: cycle: 0.5; amplitude: 20%; time: 10 min).

Polyethylene (PE) films were covered using Unicoater 409 (Erichsen, Hemer, Germany) at a temperature of 25 °C with a roller at a diameter of 40 μm. The coatings were dried for 10 min at a temperature of 50 °C. 1.6 g layers of MHPC per 1 m^2^ of PE were obtained. The active coatings contained 0.032 g of ZnO AA 44,899 particles per 1 m^2^ of PE film. PE films that were not covered were control samples (K). PE films with MHPC coatings were also used as control samples (MHPC).

The film samples were cut into square shapes (3 cm × 3 cm). The antimicrobial properties of non-covered and covered films were carried out according to ASTM E 2180-01 standard [35].

### 4.3. Accelerated Irradiation

The non-covered and covered film samples were cut into rectangle shapes (23.5 cm × 7.0 cm and 26.0 cm 2.5 cm) respectively. The samples were introduced into a UV-A accelerated weathering tester with 1.55 W/m^2^ (QUV/spray, Q-LAB) and into Q-SUN accelerated Xenon Test Chamber with 1.5 W/m^2^ (Model Xe-2, Q-LAB) and irradiated 24 h [36].

### 4.4. FT-IR

Fourier transform infrared (FT-IR) spectrum of the non-covered and covered film samples was measured using a FT-IR spectroscopy (Perkin Elmer Spectrophotometer, Spectrum 100, Waltham, MA, USA), operated at a resolution of 4 cm^−1^, over four scans. Film samples were cut into square shapes (2 cm × 2 cm) and placed directly at the ray-exposing stage. The spectrum was recorded at a wavelength of 650–4000 cm^−1^.

### 4.5. SEM

A microscopic analysis was performed using a microscope Vega 3 LMU (Tescan, Brno-Kohoutovice, Czech Repuplic) scanning electron microscope (SEM). The tests were necessary to determine the influence of accelerated irradiation on the coatings. An analysis was performed at room temperature with tungsten filament, and an accelerating voltage of 20 kV was used to capture SEM images for both the non-covered and covered films. All specimens were viewed from above.

### 4.6. Statistical Analysis

The statistical significance was determined using an analysis of variance (ANOVA) followed by a Duncan’s test. The values were considered as significantly different when *p* < 0.05. All analyses were performed with Statistica version 10 (StatSoft Polska, Kraków, Poland).
